# Assessment of Knowledge on the Prevention of Central-Line-Associated Bloodstream Infections among Intensive Care Nurses in Poland—A Prospective Multicentre Study

**DOI:** 10.3390/ijerph182312672

**Published:** 2021-12-01

**Authors:** Danuta Dyk, Agata Matusiak, Edyta Cudak, Aleksandra Gutysz-Wojnicka, Wioletta Mędrzycka-Dąbrowska

**Affiliations:** 1 Department of Anaesthesiology and Intensive Care Nursing, Medical University in Poznan, 60-179 Poznan, Poland; dyk@ump.edu.pl (D.D.); matusiak.agata25@gmail.com (A.M.); edytacud@ump.edu.pl (E.C.); 2 School of Public Health, Collegium Medicum, University of Warmia and Mazury in Olsztyn, 10-561 Olsztyn, Poland; a.gut.wojnicka@uwm.edu.pl; 3 Department of Anesthesiology Nursing & Intensive Care, Faculty of Health Sciences, Medical University of Gdansk, 80-211 Gdańsk, Poland

**Keywords:** prevention of central-line-associated bloodstream infections, intensive care nurses, evidence-based guidelines

## Abstract

The presence of a central venous catheter (CVC) leads to a high risk of blood infections, which are considered major causes of morbidity, mortality and high medical costs. The aim of this study was to assess the knowledge of nursing staff working in intensive care units (ICUs) regarding the prevention of central-line-associated bloodstream infections (CLABSIs). A nationwide survey was conducted among ICU nurses from August 2016 to April 2017. A modified Polish version of the questionnaire developed by Labeau et al. was used to assess the nurses’ knowledge. Of the 750 questionnaires distributed, 468 were returned. Women accounted for 95.73% of all respondents, and over 80% were university educated. Most of the nurses surveyed (85.9%) had previously received training in CVC guidelines, and thus over 82% rated their knowledge as good or very good. The guidelines introduced in hospitals were the main declared sources of information. In addition, more than half (68%) of respondents also knew the international guidelines. The knowledge of nursing staff in the study area is not sufficient. Studies show that the guidelines for the prevention of CLABSIs in ICUs should be standardized, and continuous training of personnel in this field should be provided.

## 1. Introduction

According to the World Health Organization (WHO), up to 30% of patients in intensive care units (ICUs) acquire healthcare-associated infections, the majority of which are bloodstream infections resulting from the presence of a catheter in the lumen of the veins. Educating staff on aseptic care of patients with a central venous catheter (CVC) can reduce the incidences of infection [1,2]. Sources indicate that nurses who receive regular training in the care of patients with a CVC demonstrate better knowledge in this area [3,4]. Moreover, nursing staff who improve their qualifications (higher education, courses, specializations) demonstrate better knowledge of vascular-line care and adherence to procedures, which significantly reduces the risk of spreading nosocomial infections [4,5].

Numerous surveys of knowledge on central-line-associated bloodstream infections (CLABSIs) involving both physicians and nurses show that nurses are more knowledgeable in this area [4,6,7,8]. On the other hand, their knowledge is unstructured.

In 2011 (edited in 2017), the Centers for Disease Control and Prevention (CDC) issued global recommendations on CVC care and the prevention of CLABSIs [9]. Based on these recommendations, internal recommendations for hospitals were developed by medical societies. The Polish version of the recommendations was published by the Polish Society of Anaesthesiology and Intensive Care in collaboration with microbiologists and epidemiologists [8] in 2013 and was adopted by the Polish Society of Anaesthesia and Intensive Care Nurses in 2018. By adhering to the above recommendations, the risk of CLABSI can be significantly reduced [1,4,10,11,12]. According to studies of Gupta et al. the introduction of a package of preventive measures resulted in the elimination of CLABSI. The number of CLABSI cases reported in 2015 and 2016 was 4 and 6, respectively, which was reduced to 0 in 2017, 2018 and 2019—with a single case in December 2018. The CLABSI rate per 1000 patient-days dropped from 3.1 per 1000 device-days to 0.4 per 1000 device-days [13].

The aim of this study was to assess the knowledge of nursing staff working in ICUs on the prevention of CLABSI.

## 2. Materials and Methods

### 2.1. Design

This study was conducted continuously from August 2016 to April 2017 and was prospective in nature. The survey questionnaire was designed for ICU nursing staff.

### 2.2. Participants

The questionnaire was distributed by the authors in hospitals in different Polish cities. Indirectly, the survey was also distributed by the provincial consultants in the anaesthesia and intensive care nursing departments in their regions. Of the 750 questionnaires distributed, 468 were returned.

### 2.3. Questionnaires and Data Collection

To assess the nurses’ knowledge, the Polish version of the questionnaire developed by Labeau et al. [14], who authorized the use of the questionnaire, was applied. The Polish version was modified at the Department of Anaesthesiology and Intensive Care Nursing at the Poznań University of Medical Sciences, where it was first used for research purposes from December 2012 to June 2013 [5].

The questionnaire had two parts. Part one consisted of questions on demographic characteristics and other independent variables. Part two consisted of 11 multiple choice questions. Correct answers were established according to the recommendations issued by the CDC and Polish recommendations [8,9]. For each correct answer, 1 point was awarded (max. 11, min. 0). The difficulty level of a question was assessed by the percentage of correct answers given. The interpretation of the results was the same as in the original questionnaire [14].

The difficulty level of the questionnaire was presented in two ways: first by using the mean, which is a popular form of data presentation in questionnaire-based research; second, it was presented using the median, because the difficulty level was a variable measured on an ordinal scale. The authors of the questionnaire considered that the median (from the second part of the questionnaire) correctly reflected nursing staff knowledge of the 11 selected evidence-based procedures used in CVC [14].

### 2.4. Ethical Aspects

The study was reviewed and approved by the Bioethics Committee of the Poznan University of Medical Sciences (resolution no. 1123/12 of 6 December 2012).

### 2.5. Statistical Analyses

For statistical analyses, descriptive statistics were used for continuous data, that is, the mean, median, minimum, maximum, lower and upper quartile, standard deviation, and Spearman’s correlation coefficient were calculated. Student’s *t*-test, Kruskal–Wallis test, and the Mann–Whitney U test were used to compare the differences between groups for continuous-type data. The chi-square test was used to examine the relationship between categorical variables. In all cases, *p* = 0.05 was established as the significance level.

## 3. Results

### 3.1. Characteristics of the Study Group

During the study, 784 questionnaires were distributed to nurses working in ICUs. The results represent information collected from 468 questionnaires, resulting in an overall response rate of 60.0%. The main demographic characteristics of the study group were presented, in which the majority were female (95.7%). Due to the purpose of the study, only diplomas of specialization in anaesthesia and intensive care nursing, as well as qualification courses in anaesthesia and intensive care nursing, were considered valid postgraduate training. Regarding the frequency of training, only 36.75% of all respondents had the opportunity to attend relevant training at least once a year, and almost 18% had never had such an opportunity. In most cases (82%), they declared that their knowledge of CVC care (before answering the 11 questions in part two of the questionnaire) was good or very good. The results also indicate that the nurses mainly learned the basic recommendations for CLABSI prevention from written hospital policy (92.09%) and international guidelines (67.95%) (Table 1).

### 3.2. ICU Nurses’ Knowledge of CLABSI Outcomes

Questions 1, 4, and 10 had the highest percentage of correct answers, while questions 5, 7, and 9 had the lowest (Table 2).

### 3.3. ICU Nurses’ Knowledge of Evidence-Based CLABSI Prevention in Relation to Demographic and Other Variables

Respondents with significant postgraduate training (course/specialization in anaesthesia and intensive care nursing) gave significantly fewer correct answers. Surprisingly, the nurses who stated that the hospital they work in did not have a standard for the management of central catheters gave more correct answers. However, the sheer number of these nurses may have influenced the result: 45 out of 468 respondents (Table 3).

The ability to attend training related to infusion therapy delivery in the workplace at least once a year significantly influenced the number of correct responses, compared to nurses who had the opportunity to train once every 1–3 years or never. Only 83 nurses had never been trained in infusion therapy management and approximately one-third of nurses received such training at least once a year. The main stated source of knowledge in this regard was hospital guidelines (92.09%).

When comparing nurses’ education, it can be seen that for those with a higher education (bachelor’s and master’s degrees), the questions proved to be easier than for the nurses who graduated from medical high school or medical vocational school (Student’s *t*-test = −3.097690, *p* = 0.00206). The mean level of difficulty for the nurses with a high school education was 0.59, while that for those with a university education was 0.64.

Statistically significant differences in responses between nurses with university education and those with medical high school or medical vocational school education were noted for questions 1, 3, 4, 7, and 9 (*p* < 0.05). For questions 1, 3, 4, and 9, more correct answers were given by nurses with bachelor’s and master’s degrees, while question 7 was better answered by nurses without a university degree (Table 4).

In the case of questions in which there were statistically significant differences in the answers, excluding question 7, the group of people with a university education obtained better results than the high school group.

### 3.4. CLABSI Prevention Behaviours

Only 7 out of the 468 nurses surveyed said that CVCs are changed routinely every 7 days, while the vast majority answered correctly that CVCs are changed only when indicated. Antiseptic-coated central catheters, as recommended, are used for patients in whom the catheter will stay in the vessel for more than 5 days, as indicated by 56% of respondents. Regarding dressing change, the vast majority of nurses correctly indicated that a dry, clean, transparent dressing is changed every 7 days, while a dry and clean gauze dressing should be changed every 2 days, as 87% of nurses believe. One of the most difficult questions was the question regarding the recommended protection of the injection site. Most nurses stated that only a polyurethane, transparent dressing is required; however, according to the recommendations, both a transparent dressing and a gauze dressing are recommended to protect the CVC insertion site.

The recommended disinfectant is a 0.5–2% alcohol solution of chlorhexidine gluconate, and this was indicated by almost 58% of the nurses surveyed. The application of antibiotic ointment to the insertion area is not recommended because it generates the emergence of antibiotic resistance, but only 27% of nurses answered correctly. Replacement of lipid transfusion sets should properly take place within 24 h, while the transfusion of one unit of blood and blood products can only be done through one transfusion set, which 384 nurses were aware of. Intermittent transfusion sets, on the other hand, are replaced within 24 h, and this is what nearly 95% of respondents indicated.

The last question asked about disinfecting the outlet of a catheter or connector to a CVC before connecting it to the kit. Wiping with a 70% alcohol solution or an alcoholic chlorhexidine solution for no less than 15 s is recommended. The correct answer was indicated by 61% of the nurses, and 36% only sprayed the outlet with the same solution. Comparing the subjective assessment of the studied group of nurses to the correct answers given in the questionnaire, no significant statistical relationship was observed (Kruskal–Wallis test: H (4, N = 468) = 3.050160, *p* = 0.5495). Nurses who were unsure whether their answers were correct responded similarly to those who were completely sure (Table 5).

## 4. Discussion

The presence of a CVC places patients at high risk of blood infections, which are considered major causes of morbidity, mortality and high medical costs [4]. In Poland, it is assumed that approximately 0.5 million patients develop a nosocomial infection, which could mean that approximately 50,000 patients each year develop an infection related to vascular-line and intravenous therapy [15]. According to more recent estimates, which are still based only on the incidence of CLABSIs/1000 catheter-days in other countries, it may be assumed that, in Poland, 5000–10,000 cases of CLABSI occur every year, particularly in ICUs [16,17].

In the first edition of the same survey, nurses with “substantial” postgraduate training gave significantly more correct answers (mean—0.55), compared to those without training (mean—0.46). In contrast, there was no statistically significant difference in the second edition. However, in both editions, age and length of service had no effect on nurses’ knowledge of CLABSI. In addition, nurses with a university education (bachelor’s or master’s degree) found the questions easier than nurses who graduated from medical high school or medical vocational school [15]. More correct answers were given by nurses in the second edition. In terms of self-assessment, however, nurses who rated their knowledge as inadequate actually gave significantly fewer correct answers, but only in the first edition. In a later study, nurses who were unsure if their answers were correct responded similarly to those who were completely sure. A similar analysis showed that nurses rated their knowledge at a high level, but this was not reflected in the number of correct answers they gave [15]. Another study indicated that the lower the level of knowledge demonstrated by the nurse, paradoxically, the higher their self-esteem was, which the authors explain is due to the excessive routine of work [18].

Nurses have been the main study group in most studies assessing the knowledge of healthcare personnel regarding blood infections [4]. They are the ones who provide constant care to patients and play a vital role in the care and maintenance of vascular catheters, which explains why they have shown much better knowledge in this area than physicians. However, even this knowledge is insufficient and needs to be standardized [4,6,7]. Studies have shown that systematic, brief training of nursing staff increases their knowledge and thus reduces the incidence of CLABSI [17] by up to 41.7% [1]. Out of 27 studies, only one did not show a reduction in CLABSI cases after adequate educational training had been provided [4]. Further research into the education of staff (both nurses and physicians) and its impact on the spread of nosocomial infections, as well as the provision of regular training, appears to be necessary. However, it is difficult to determine the most effective method of education due to the existence of different approaches. For instance, Almahmoud et al. pointed out that although adherence to the guidelines is high, training that focuses on individual, less adhered to recommendations would be advisable [19]. Manzo et al. drew a similar conclusion from their study of professionals in neonatal and paediatric units [20]. Additionally, Esposito et al. clearly saw the need to include the current evidence-based practice guidelines in the educational curricula for healthcare workers [21].

## 5. Strengths and Limitations

The assessment of knowledge and behaviour through self-description, and not through observation is a limitation of this study. When planning future research, it is worth considering the use of a direct observation checklist in order to reduce bias. In addition, this was a cross-sectional study, which makes it impossible to determine the temporality of the associations presented in the results.

## 6. Conclusions

The knowledge of nursing staff in the study area is not sufficient. Studies show that the guidelines for the prevention of CLABSIs in ICUs should be standardized, and continuous training of personnel in this field should be provided.

## 7. Practical Implications of the Study

The study reflects the disparity between what is considered an appropriate course of practice and the lack of consistent prevention of body substance isolation (BSI) related to CVCs.

## Figures and Tables

**Table 1:** Characteristics of participants in the whole group.

Variable	Value (N = 468)
Sex	
Female	448 (95.73%)
Male	20 (4.27%)
Age (years)	46.56 ± 9.46 SD
Years of practice	17.81 ± 10.82 SD
Education	
Medical high school	55 (11.75%)
Medical professional study	38 (8.12%)
Bachelor’s degree	123 (26.28%)
Higher education	252 (53.85%)
Important postgraduate training	
Yes	402 (85.90%)
No	66 (14.10%)
CVC maintenance hospital written policy	
Yes	423 (90.38%)
No	45 (9.62%)
I do not know	0 (0.00%)
Accessibility of training	
At least once a year	172 (36.75%)
Every 1–3 y	71 (15.17%)
Rarely	142 (30.34%)
Never	83 (17.74%)
Self-assessment	
Very good	126 (26.92%)
Good	260 (55.56%)
Satisfactory	68 (14.53%)
Unsatisfactory	14 (2.99%)
Sources of knowledge *	
Hospital written policy	431 (92.09%)
International written policy	318 (67.95%)
National written policy	189 (40.38%)
Course conferences on the subject	183 (39.10%)

Note: Values are N (%) or mean ± SD. CVC, central venous catheter. * The total may not sum to the total number because >1 source of the relevant knowledge was given.

**Table 2:** Responses to each question given by the participants.

Questions	Respondents	Difficulty
1. It is recommended to replace CVCs routinely.		0.99
(A) Yes, every 7 d	7 (1.50%)	
(B) Yes, every 3 d	0 (0.00%)	
(C) * No, only when indicated	461 (98.50%)	
(D) I do not know	0 (0.00%)	
2. In settings with a high rate of CLABSI, it is recommended to use a CVC coated or impregnated with an antiseptic agent.		0.56
(A) * Yes, in patients whose CVC is expected to be remained in place for >5 d	264 (56.41%)	
(B) No, because the use of such catheters is not cost-effective	99 (21.15%)	
(C) No, because the use of such catheters does not result in a significant decrease in the rate of catheter-related infections	17 (3.63%)	
(D) I do not know	88 (18.81%)	
3. It is recommended to change the clean, dry, and intact transparent dressing on the catheter insertion site.		0.73
(A) Every 2 d	76 (16.24%)	
(B) Every 5 d	39 (8.33%)	
(C) * Every 7 d	343 (73.29%)	
(D) Only when the catheter is replaced	10 (2.14%)	
4. It is recommended to change the clean, dry, and intact gauze dressing on the catheter insertion site.		0.87
(A) * Every 2 d	408 (87.18%)	
(B) Every 5 d	12 (2.56%)	
(C) Every 7 d	32 (6.84%)	
(D) Only when the catheter is replaced	13 (2.78%)	
(E) I do not know	3 (0.64%)	
5. It is recommended to cover up the catheter insertion site with		0.18
(A) Polyurethane dressing (transparent, semipermeable)	374 (79.91%)	
(B) Gauze dressing	9 (1.92%)	
(C) * Both are recommended because they do not affect the risk of catheter-related infections	84 (17.95%)	
(D) I do not know	1 (0.22%)	
6. It is recommended to disinfect the catheter insertion site with		0.58
(A) 2% aqueous chlorhexidine	161 (34.40%)	
(B) * 0.5%–2% alcoholic gluconate chlorhexidine	270 (57.69%)	
(C) 10% povidone-iodine	15 (3.21%)	
(D) I do not know	22 (4.70%)	
7. It is recommended to apply an antibiotic ointment at the insertion site of a CVC.		0.28
(A) Yes, because it decreases the risk of catheter-related infections	132 (28.21%	
(B) * No, because it causes antibiotic resistance	129 (27.56%)	
(C) No, because it does not decrease the risk of catheter-related infections	178 (38.33%)	
(D) I do not know	29 (6.20%)	
8. When (1) lipid emulsions or (2) blood and blood products are administered through a CVC, it is recommended to replace the administration set.		0.82
(A) (1) Every 12 h, (2) 4 blood units or blood components can be administered through 1 administration set	63 (13.46%)	
(B) * (1) Within 24 h, (2) 1 blood unit or blood component can be administered through 1 administration set	384 (82.06%)	
(C) (1) Every 72 h, (2) 2 blood units or blood components can be administered through 1 administration set	7 (1.49%)	
(D) I do not know	14 (2.99%)	
9. When liquids other than blood, blood products, or fat emulsions are administered continuously, the administration set should be replaced.		0.34
(A) Every 48 h	288 (61.53%)	
(B) * Every 72–96 h	154 (32.91%)	
(C) Every 96 h	5 (1.07%)	
(D) I do not know	21 (4.49%)	
10. Administration sets used in intermittent infusion (when bottles with liquids are connected and disconnected for each dose) should be replaced.		0.95
(A) * Every 24 h	444 (94.88%)	
(B) Every 72 h	17 (3.63%)	
(C) Every 96 h	1 (1.28%)	
(D) I do not know	6 (0.21%)	
11. It is recommended to use an antiseptic agent to clean the access hub or connector before the connection of the administration set or after unscrewing the dead-end cap which closes the catheter.		0.61
(A) * Yes, by wiping with 70% alcohol solution or alcohol and chlorhexidine solution for no less than 15 s	286 (61.11%)	
(B) Yes, by spraying the access site with 70% alcohol solution or alcohol chlorhexidine solution	169 (36.11%)	
(C) It is not recommended because no evidence has been found for the relationship between the disinfection of the connecting site of the administration set and the contamination of fluids in the insertion hub	4 (0.86%)	
(D) I do not know	9 (1.92%)	

Note: Values are n (%) or as otherwise indicated. CVC, central venous catheter. * Correct answer.

**Table 3:** Descriptive statistics of correct and incorrect answers according to the variables.

Differences and Relations among the Selected Variables and Knowledge of Evidence-Based Practices for Preventing CLABSI								
Variable	N	Mean	Median	Min.	Max.	Lower Quartile	Upper Quartile	*p*-Value
Difficulty of the questionnaire	468	0.629	0.636	0.273	1.000	0.545	0.727	
Important postgraduate diploma training								0.0279
Yes	402	0.591	0.545	0.273	1.000	0.455	0.727	
No	66	0.554	0.545	0.273	0.818	0.455	0.636	
Standard maintenance CVC								<0.001
Yes	423	0.570	0.545	0.273	1.000	0.455	0.636	
No	45	0.727	0.727	0.727	0.727	0.727	0.727	
Accessibility of training								<0.001
At least once a year	172	0.636	0.727	0.364	1.000	0.545	0.727	
Every 1–3 y	71	0.576	0.545	0.273	0.818	0.545	0.636	
Rarely	142	0.546	0.545	0.273	0.818	0.455	0.636	
Never	83	0.555	0.545	0.273	0.818	0.455	0.636	
Age (years)								0.11
Years of practice								0.08

CVC, central venous catheter; ICU, intensive care unit.

**Table 4:** Comparison of the results of answers to questions regarding the level of education.

Question	Chi-Square Test	*p*-Value
1. It is recommended to replace CVCs routinely.	6.199605	*p* = 0.01278
2. In settings with a high rate of CLABSI, it is recommended to use a CVC coated or impregnated with an antiseptic agent.	1.627868	*p* = 0.20200
3. It is recommended to change the clean, dry, and intact transparent dressing on the catheter insertion site.	20.18677	*p* = 0.00001
4. It is recommended to change the clean, dry, and intact gauze dressing on the catheter insertion site.	4.433834	*p* = 0.03523
5. It is recommended to cover up the catheter insertion site with	2.006253	*p* = 0.15665
6. It is recommended to disinfect the catheter insertion site with	0.1503781	*p* = 0.69817
7. It is recommended to apply an antibiotic ointment at the insertion site of a CVC.	4.703333	*p* = 0.03010
8. When (1) lipid emulsions or (2) blood and blood products are administered through a CVC, it is recommended to replace the administration set.	0.9969262	*p* = 0.31806
9. When liquids other than blood, blood products, or fat emulsions are administered continuously, the administration set should be replaced.	4.420548	*p* = 0.03551
10. Administration sets used in intermittent infusion (when bottles with liquids are connected and disconnected for each dose) should be replaced.	0.0146888	*p* = 0.90353
11. It is recommended to use an antiseptic agent to clean the access hub or connector before the connection of the administration set or after unscrewing the dead-end cap which closes the catheter.	0.0768563	*p* = 0.78160

**Table 5:** Comparison of self-assessment of nurses to the percentage of correct answers.

Self-Assessment	Percentage of Correct Answers
Completely unsure	0.545455
Unsure	0.600000
Neutral	0.618538
Sure	0.631957
Completely sure	0.560606

## Data Availability

The authors declare that the data of this research are available from the corresponding author on request.
